# Case report: Robot-assisted laparoscopic partial nephrectomy for renal cell carcinoma in a patient with situs inversus totalis and abdominal cocoon

**DOI:** 10.3389/fsurg.2023.1095591

**Published:** 2023-02-17

**Authors:** Yuhua Zou, Xiaojuan Xie, Cunzhi Zhong, Li Liu, Qinlin Wang, Sheng Yan, Xiaofeng Zou, Quanliang Liu

**Affiliations:** ^1^ Department of Urology, The First Affiliated Hospital of Gannan Medical University, Ganzhou, China; ^2^ Department of Cardiology, The First Affiliated Hospital of Gannan Medical University, Ganzhou, China; ^3^ Department of Anesthesiology, Operation Rom, The First Affiliated Hospital of Gannan Medical University, Ganzhou, China

**Keywords:** situs inversus totalis, abdominal cocoon, renal cell carcinoma, robot-assisted laparoscopic, partial nephrectomy, case report

## Abstract

**Background:**

Situs inversus totalis (SIT) is a congenital condition wherein organs in abdominal or thoracic cavity are mirrored from their normal positions. Abdominal cocoon, is a rare disease of unknown aetiology that is characterised by total or partial small intestine encapsulation by a compact fibrocollagenous membrane. Aside from having two extremely rare conditions (SIT and Abdominal cocoon), our patient developed renal cell carcinoma (RCC), which makes this case even more uncommon.

**Case Presentation:**

We report the case of a 64-year-old man who was admitted to our hospital with an extremely rare case of localized RCC in the left kidney complicated with SIT and abdominal cocoon. Computer tomography urography (CTU) and angiography (CTA) showed that the patient was confirmed as having SIT, for the space-occupying lesion in the left kidney, clear cell RCC (ccRCC) was considered, the lesion in the right kidney was probably cystic. We diagnosed our patient as having a cT1aN0M0 left RCC, and the RENAL score was 7x. With partial nephrectomy (PN) being the preferred treatment approach, robot-assisted laparoscopic partial nephrectomy (RALPN) was performed after obtaining informed consent. After insertion of the laparoscope, adhesions were observed between the entire colon and the anterior abdominal wall. Then, abdominal cocoon was diagnosed. The surgery was uneventful, and the tumour was resected successfully while preserving the tumour capsule. No intestinal injury or any other complication occurred in the intraoperative or postoperative, and the patient recovered well after the operation.

**Conclusion:**

PN is an extremely challenging procedure in patients with SIT and abdominal cocoon. The da Vinci Xi surgical system and thorough preoperative assessment allowed the surgeon to overcome stereotyping, visual inversion, and successfully perform PN in a patient with SIT and abdominal cocoon without increasing the risk of complications and preserving as much renal function as possible. Considering the satisfactory outcomes, this report may hopefully provide a practical reference for the treatment of RCC in patients with other special conditions.

## Introduction

Situs inversus totalis (SIT) is a congenital condition wherein organs in the abdominal or thoracic cavity are mirrored from their normal positions. This rare anatomical anomaly has an incidence of approximately 0.005%–0.01% (1). Abdominal cocoon, which is also known as idiopathic sclerosing peritonitis, congenital small intestinal obstruction and fibromembranous encapsulation, is a rare disease of unknown aetiology that is characterised by total or partial small intestine encapsulation by a compact fibrocollagenous membrane (2).

Renal cell carcinoma (RCC) is a malignancy of the urinary system that accounts for 2%–3% of all adult malignancies (3). For patients with T1a RCC, partial nephrectomy(PN) is recommended (4). This report presents an extremely rare case of localized RCC in the left kidney in a patient with SIT and abdominal cocoon, and aims to address gaps in treating RCC with robot-assisted laparoscopic partial nephrectomy (RALPN) in these patients. In patients with SIT and abdominal cocoon, surgery may be extremely difficult as SIT is complicated by structural deformities, and lysis of extensive intestinal adhesions may cause secondary intestinal injury. In our case, the da Vinci Xi surgical system enabled successful RALPN of the left kidney, which not only avoids the unclear exposure of the mirror human anatomical structure under traditional laparoscopic surgery, but also solves the problem of the operator's right hand inversion during the operation.

## Case presentation

**Figure 1:**
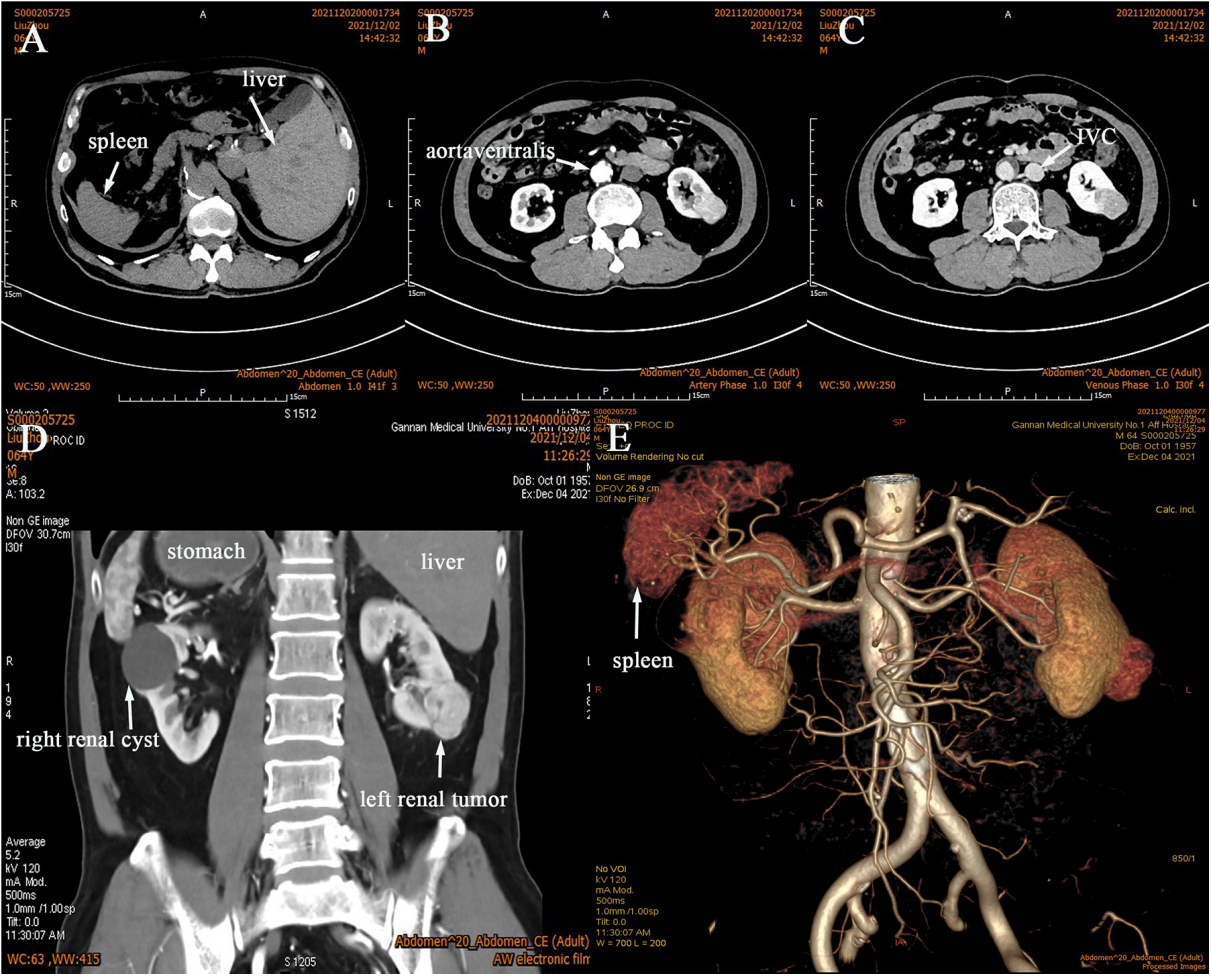
CTU and CTA was performed, and it confirmed SIT (**A–E**) with a tumor in the left kidney (**B–E**). It showed that the left renal artery had anatomical variation (**E**), the arterial phase (**B**), the venous phase (**C**), the coronal view (**D**).

The reporting of this study conforms to CARE guidelines (5). In Dec. 3, 2021, a 64-year-old man with a solid space-occupying lesion that measured 25 × 36 mm in the inferior pole of the left kidney by Doppler ultrasound during physical examination one month prior. The body mass index (BMI) was 22.19 kg/m². Meanwhile, the patient was generally well, was afebrile, and did not experience low back pain or discomfort, frequent urination, urgency, painful urination and gross haematuria. Additionally, the patient denied gastrointestinal symptoms such as abdominal pain and distention, nausea and vomiting. The patient had a 6-year special history of hypertension, and his blood pressure was well controlled by regular oral antihypertensive drugs. During hospitalisation, his maximum monitored blood pressure (BP) was 135/97 mmHg, and his monitored heart rate ranged from 72 to 90 bpm. The patient denied any history of other medications, diabetes, abdominal surgery or trauma, abdominal tuberculosis, peritoneal dialysis, autoimmune disease, or chemotherapy. Preoperative blood tests and comprehensive metabolic panel suggested no abnormalities. Renal Doppler ultrasound showed a solid space-occupying lesion in the left kidney, which required further evaluation. Computer tomography urography (CTU) and angiography (CTA) showed that the stomach and spleen were located in the right abdominal cavity, while the right hepatic lobe, gallbladder and inferior vena cava were at the left side of his abdomen (Figures 1A–E), the bilateral kidneys were normal in size and shape, but a 24 × 35 mm isodense mass protruded from the outline of the left kidney (Figures 1B–E). On contrast-enhanced computed tomography (CT) scan, the mass was strongly enhanced in the arterial phase (Figure 1C), and the enhancement pattern was attenuated during the venous phase (Figure 1D). There was a 31 × 40 mm non-enhancing hypodense nodule and calcification of the wall in the right renal. Additionally, the left renal artery was supplied by one renal artery and one accessory renal artery (Figure 1E). Effusion or lymphadenopathy in the abdominopelvic cavity was not noted. Based on the above findings, the patient was confirmed as having SIT, for the space-occupying lesion in the left kidney, clear cell RCC (ccRCC) was considered, the lesion in the right kidney was probably cystic. Cardiac Doppler ultrasound demonstrated decreased diastolic function and normal systolic function of the left ventricle in the transposed heart. The estimated glomerular filtration rates (eGFR) were 29.76 and 28.8 ml/min for the left and right kidneys, respectively. Chest CT showed no space-occupying lesions or evident abnormalities. Before surgery, the patient was diagnosed with left RCC, SIT, right renal cyst and hypertension. We diagnosed our patient as having a cT1aN0M0 left RCC, and the RENAL score was 7x. With PN being the preferred treatment approach, left RALPN was performed after obtaining informed consent.

**Figure 2:**
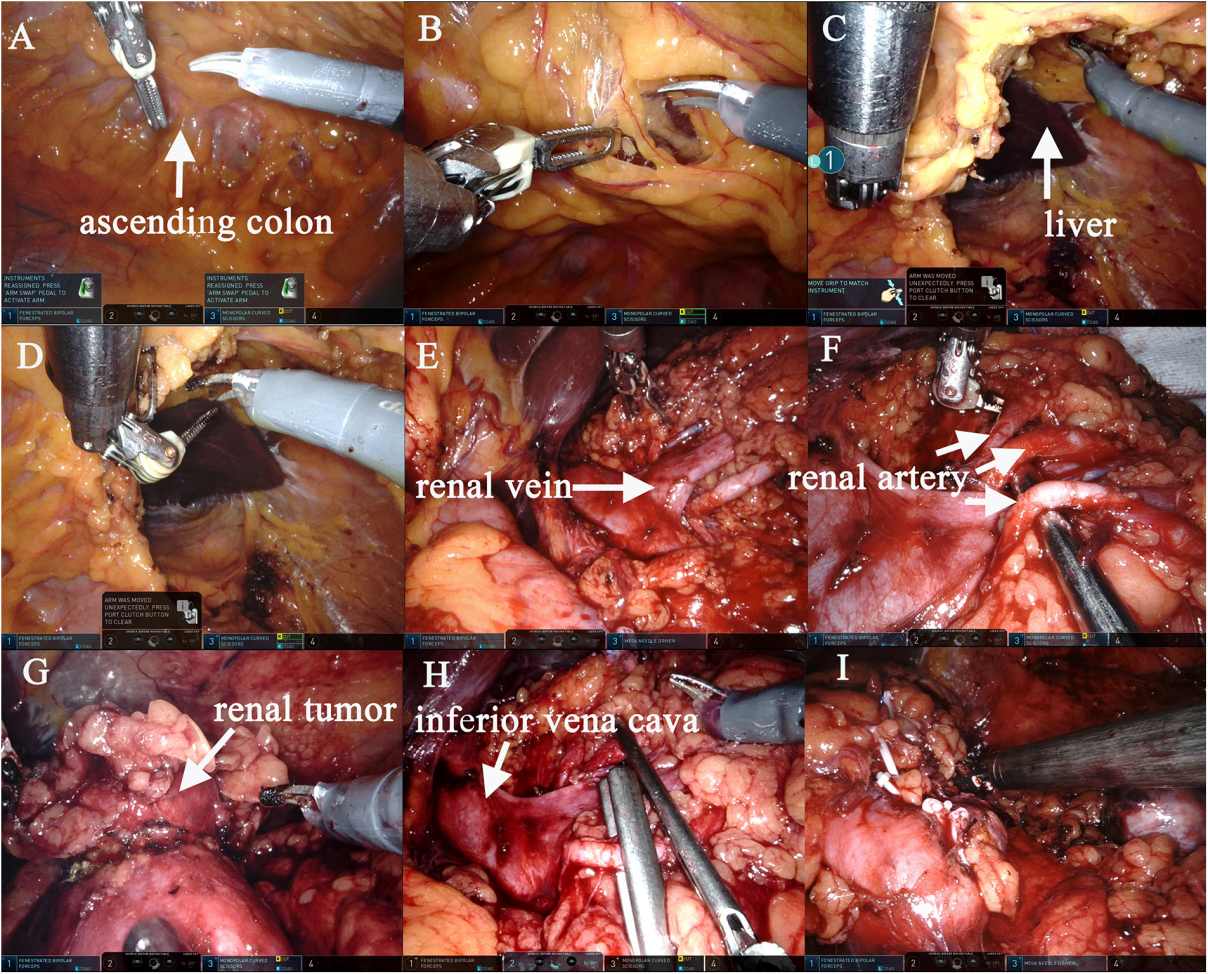
The left RALPN was performed. Adhesions were observed between the entire colon and the anterior abdominal wall, and the liver and the visual field of the surgical area could not be visualized (**A,B**). After adhesions between the colon and the anterior abdominal wall were removed, the liver and kidney areas on the left were exposed (**B–D**). The inferior vena cava was located on the left side (**E,H**), accurate dissection of the left renal hilar vessels (**E**), three arterial branches were dissected (including one from the abdominal aorta to the lower pole of the kidney, and two from the main renal artery to the middle and lower poles of the kidney) (**F**). The tumor was isolated and the margin was marked (**G**). Two branched renal arteries were blocked (**H**). The tumor was completely removed and the left renal margin was sutured accurately (**I**).

**Figure 3:**
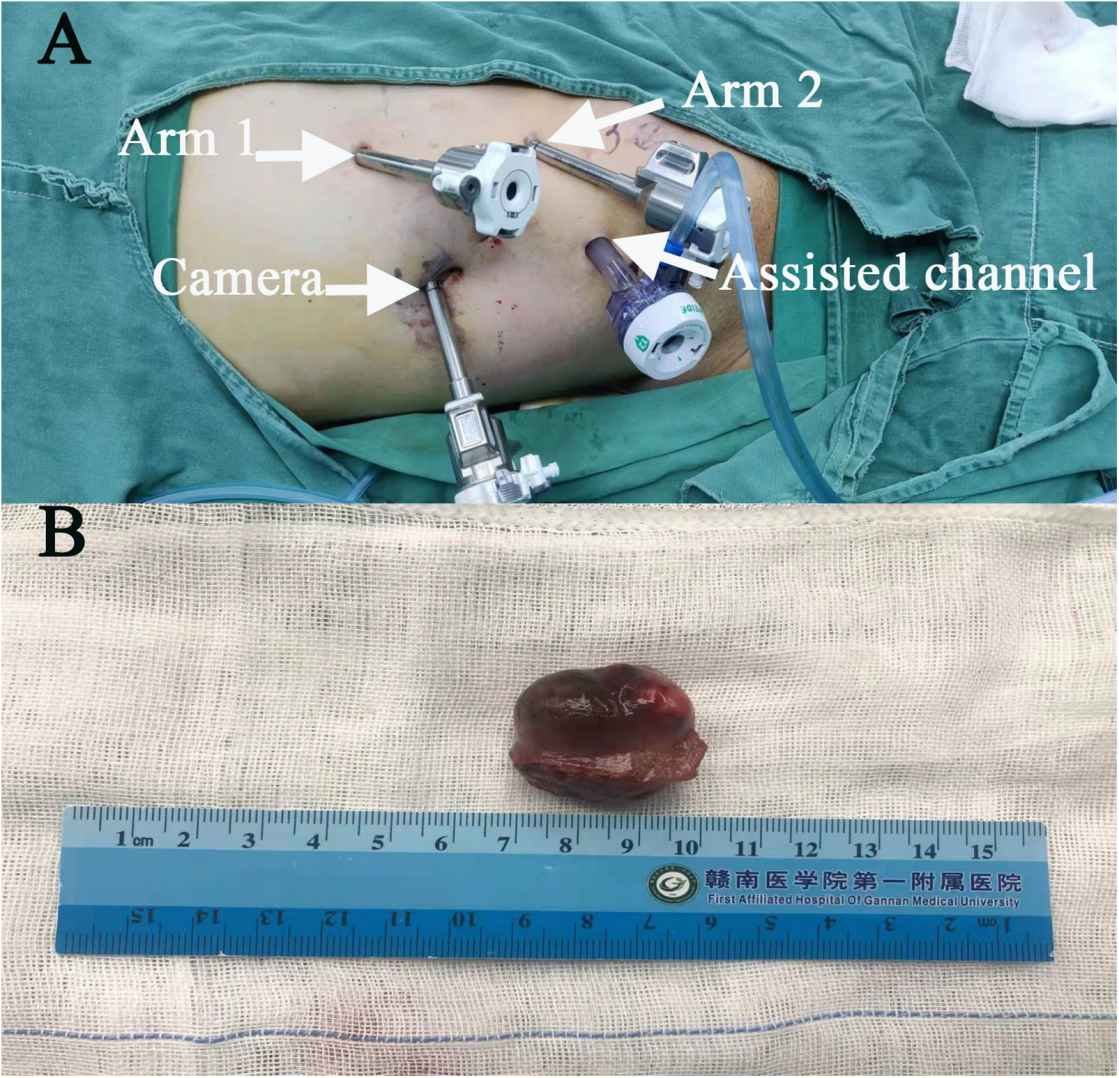
The distribution of ports in this case (**A**). Specimen: tumour capsule integrity (**B**).

**Figure 4:**
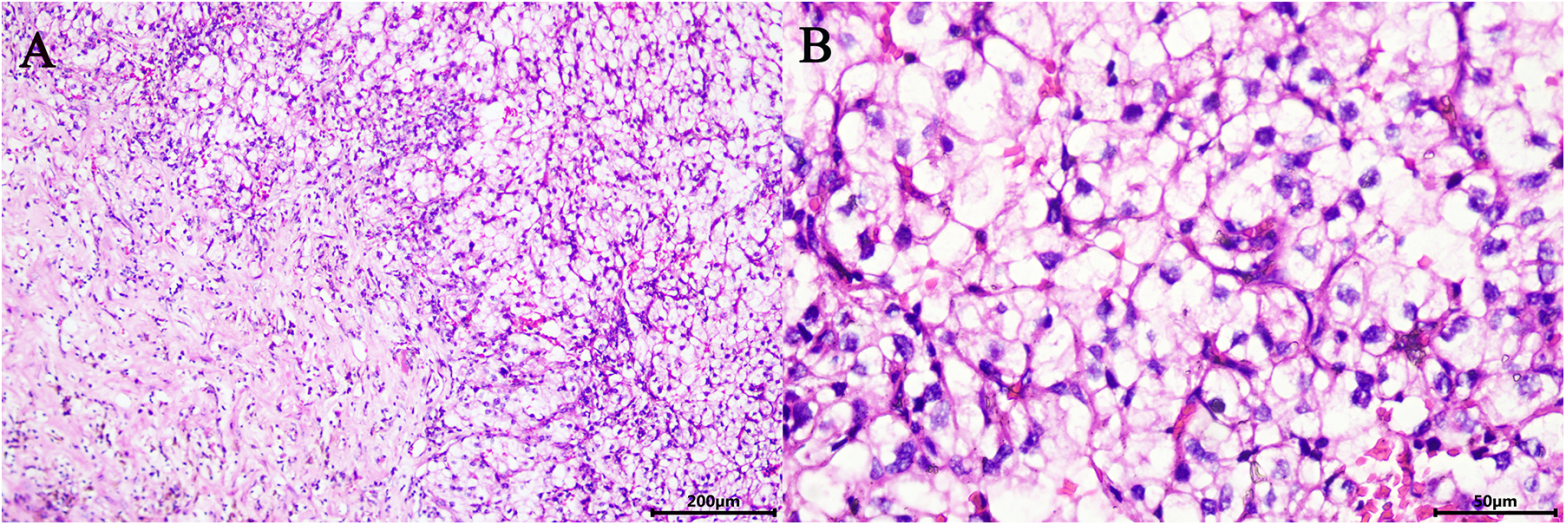
Microscopic appearance (**A,B**). Pathology revealed pT1aN0M0 Fuhrman Grade I ccRCC (hematoxilyn and eosin staining, ×200 (**A**), ×400 (**B**)).

Surgery was performed under general anaesthesia with the patient being placed in a supine position and angled at 70° on the unaffected side. A 1.0 cm incision was created at the medial umbilical edge for insertion of a Veress needle and induction of CO₂ pneumoperitoneum that was maintained at 15 mmHg. A laparoscope was then inserted to observe the abdominal cavity, adhesions were observed between the entire colon and the anterior abdominal wall, particularly between the intestines (Figures 2A,B). Based on these findings, abdominal cocoon was diagnosed. Under direct laparoscopy, two 8-mm robotic ports were respectively placed in adhesion-free areas in the intestine 4 cm from the anterior superior iliac spine and under the costal margin (Figure 3A), while a 12-mm assistant port was inserted 5 cm under the umbilicus at the midline (Figure 3A). When the robotic laparoscopy system was ready, adhesions between the colon and the anterior abdominal wall were removed (Figure 2B–D). The transposed organs, including the liver, spleen, stomach and colon, were then noted (Figure 2C,D). The inferior vena cava was located on the left side (Figure 2E–H). A protruding tumour (Figure 2G) was found in the left kidney, three arterial branches were dissected (including one from the abdominal aorta to the lower pole of the kidney, and two from the main renal artery to the middle and lower poles of the kidney) (Figure 2F). The surgery was uneventful, and the tumour was resected successfully while preserving the tumour capsule (Figure 2I). The operation time was 180 min, which included 23 min of warm ischemia time. The intraoperative blood loss was 200 ml. A 23 × 36 mm specimen was removed from the left kidney and sectioned to confirm tumour capsule integrity (Figure 3B). Pathology revealed pT1aN0M0 Fuhrman Grade I ccRCC, which did not involve the surgical margins (Figure 4A,B). No intestinal injury or any other complication occurred in the intraoperative or postoperative. The patient was satisfied with the treatment. The patient was discharged at postoperative on day 7. Postoperatively, the patient was followed up for 10 months, and no recurrence, secondary infection, or intestinal obstruction was detected.

## Discussion

SIT is the complete transposition of both the abdominal and thoracic organs that does not affect the anatomical relationships of the adjacent organs or their physiological functions, and anatomical variation of the great vessels, organ malformations and other congenital anomalies are common in patients with SIT (1). The mechanism responsible for such reversal is still unknown. Some believe that SIT involves autosomal recessive inheritance and genetic abnormalities, while others attribute SIT to malrotation during embryonic development (1). Recently, attention has focused on the association between SIT and cancer (6). Haruki et al. reported that KIF3 complex deficiency, a congenital characteristic in patients with SIT, appears to promote cancer development and progression (7).

Abdominal cocoon is a rare abdominal disorder first described by Foo et al. in 1978 (8). The syndrome lacks distinct clinical features and thus is difficult to diagnose before surgery. Although there is evidence of potential associations between abdominal cocoon and developmental abnormalities and medication, infection and surgery history, the underlying mechanism remains unknown (9). Abdominal cocoon can either be primary or secondary. Primary abdominal cocoon is idiopathic and commonly seen in patients without a history of abdominal surgery or trauma and may be linked to congenital dysplasia of the omentum majus and peritoneum (2). Secondary abdominal cocoon has a complex aetiology associated with previous surgery, peritoneal dialysis, tuberculous disease and gastrointestinal cancer (10, 11). Abdominal cocoon has nonspecific clinical manifestations, patients can be asymptomatic in mild cases. Common symptoms include an abdominal mass, persistent abdominal pain and incomplete bowel obstruction (12). Since preoperative diagnosis of abdominal cocoon is difficult, in most reported cases, the diagnosis is established based on intraoperative findings. In this report, the radiologist did not consider the possibility of abdominal cocoon. The patient was not diagnosed with abdominal cocoon until surgery, wherein bowel adhesions involving the descending colon, small intestine and peritoneum were observed.

Aside from having two extremely rare conditions (SIT and abdominal cocoon), our patient developed RCC, which makes this case even more uncommon. It is generally believed that laparoscopic surgery is safe and feasible even for patients with SIT. In fact, this approach has already been used in surgical conditions involving the gallbladder, bile duct, stomach, kidney and liver (1, 13). While several studies have reported open/laparoscopic radical nephrectomy (RN) (14, 15) or open PN (16) in patients with SIT and renal carcinoma, no case of simultaneous abdominal cocoon and RCC has been covered in these reports. Furthermore, there are no reports of RALPN in patients with SIT and abdominal cocoon.

In this case, given the transposition of internal organs and the goal of preserving as much renal parenchyma as possible, surgery was performed by an experienced surgeon who was thoroughly familiar with abdominal anatomy. Pneumoperitoneum was established to visualise the abdominal cavity, and abdominal cocoon was considered due to bowel encapsulation. Abdominal cocoon caused difficulties for the surgeon. Fortunately, we chose the medial side of the umbilical margin to insert the pneumoperitoneum needle and camera port first, which did not cause secondary intestinal injury. Additionally, other ports were inserted under direct laparoscopy and kept away from the bowel adhesions. So the port distribution was different from the conventional one. Despite the lack of international guidelines for abdominal cocoon management, the standard treatment for the disease includes removal of the sac and adhesiolysis (17). Because the patient was asymptomatic on weekdays, adhesiolysis was performed intraoperatively to separate the intestines and the peritoneum. However, adhesiolysis does not necessarily require complete removal of the sac or lysis of the bowel loops as long as the small intestines can be released and the intestines and mesentery can return to their normal positions without inflicting secondary injury.

The presence of vascular malformations, which often occur with SIT, should be considered when performing preoperative imaging tests. For patients with SIT and surgical disease, it is important to define the mirror-image characteristics to reduce the risk of vascular abnormalities before surgery (18). In our case, CTU and CTA were performed to visualise the tumour size and location as well as to determine the spatial relationship between the tumour and adjacent tissues. Additionally, the operation was performed based on a three-dimensional (3D) reconstruction of the renal vasculature that revealed the arterial orientation and variations of the affected kidney. Complete separation of the renal arterial divisions played a crucial role in reducing intraoperative bleeding and ensuring high visibility and effective tumour control. Intraoperative findings showed that the left renal artery had anatomical variation, which was consistent with the preoperative assessment, if this was incorrect, surgery may have damaged the renal artery and injured the kidney. In patients with SIT and abdominal cocoon, RALPN is technically demanding because it requires anti-conventional thinking and heightened awareness of the mirror-image reversal of internal organs. In this report, the da Vinci Xi surgical system provided the operator with a surgical field magnified by >10-fold, high-definition 3D images, and robotic arms with seven planes of motion, and played an important role in all aspects (19), allowing for easier and safer separation of bowel adhesions, dissection of the renal hilum, separation of abnormal renal arteries, identification of surgical margins, tumour resection and wound closure with surgical precision.

## Conclusions

PN is an extremely challenging procedure in patients with SIT and abdominal cocoon. The da Vinci Xi surgical system and thorough preoperative assessment allowed the surgeon to overcome stereotyping, visual inversion, and successfully perform PN in a patient with SIT and abdominal cocoon without increasing the risk of complications and preserving as much renal function as possible. Considering the satisfactory outcomes, this report may hopefully provide a practical reference for the treatment of RCC in patients with other special conditions.

## Data Availability

The original contributions presented in the study are included in the article/Supplementary Material, further inquiries can be directed to the corresponding author/s.
